# The prognostic value of sarcopenia and sarcopenic obesity in patients with lung cancer receiving immunotherapy: a propensity score matching study

**DOI:** 10.1093/oncolo/oyaf114

**Published:** 2025-06-23

**Authors:** Wen Wang, Xintian Xu, Hongming Liu, Yongxia Cui, Qian Han, Tingting Yang, Mengxing Tian, Yu Qian, Xin Jin, Lei Lei

**Affiliations:** Department of Nutrition, Henan Provincial People’s Hospital, Zhengzhou University People’s Hospital, Zhengzhou, Henan, China; Department of Pharmacy, Hubei Cancer Hospital, Tongji Medical College, Huazhong University of Science and Technology, Wuhan, Hubei, China; Department of Imaging, Henan Provincial People’s Hospital, Zhengzhou University People’s Hospital, Zhengzhou, China; Cancer center, Henan Provincial People’s Hospital, Zhengzhou University People’s Hospital, Zhengzhou, China; Cancer center, Henan Provincial People’s Hospital, Zhengzhou University People’s Hospital, Zhengzhou, China; Department of Nutrition, Henan Provincial People’s Hospital, Zhengzhou University People’s Hospital, Zhengzhou, Henan, China; Department of Clinical Nutrition, Hubei Cancer Hospital, Tongji Medical College, Huazhong University of Science and Technology, Wuhan, Hubei, China; Department of Medical Oncology, Hubei Cancer Hospital, Tongji Medical College, Huazhong University of Science and Technology, Wuhan, Hubei, China; Department of Clinical Nutrition, Hubei Cancer Hospital, Tongji Medical College, Huazhong University of Science and Technology, Wuhan, Hubei, China; Department of Gastroenterology, Henan Provincial People’s Hospital, Zhengzhou University People’s Hospital, Zhengzhou, Henan, China

**Keywords:** sarcopenia, sarcopenic obesity, body composition, immune checkpoint inhibitors, nomogram

## Abstract

**Background:**

Sarcopenic obesity (SO) is a prognostic factor and its impact on response to immunotherapy is still unknown in lung cancer. We aimed to explore the role of SO and body composition in predicting overall survival (OS) in patients with lung cancer receiving immune checkpoint inhibitors (ICIs).

**Methods:**

We conducted a retrospective study involving 119 patients with lung cancer who underwent immunotherapy. The subcutaneous fat area (SFA), visceral fat area (VFA) and skeletal muscle index (SMI) were determined by the cross-sectional computed tomography at the L3 lumbar vertebral level. Sarcopenia and SO were defined by SMI and body mass index. Kaplan–Meier and Cox proportional hazard analyses were used to evaluate the impact of body composition on survival. The propensity score matching (PSM) analysis was used to reduce bias and a nomogram was created to predict the OS.

**Results:**

The Kaplan–Meier survival showed that patients with sarcopenia and SO had poor survival time in the total and PSM cohort. The Cox analyses revealed that sarcopenia (Hazard ratio (HR): 2.04, 95% Confidence Interval (CI): 1.04-4.01, *P* = 0.039) and SO (HR: 3.17, 95%CI: 1.49-6.75, *P* = 0.003) were independent OS predictors. SFA and VFA were not associated with OS. The SMI, age, stage, albumin level and SFA were used to develop a nomogram. Patients with high nomogram scores had worse OS (*P* < 0.0001).

**Conclusions:**

Sarcopenia and SO are prognostic factors in patients with lung cancer receiving ICIs. A nomogram that integrates body composition is sufficiently accurate for predicting OS in patients with lung cancer receiving immunotherapy.

**Implications for Practice**

The findings from our study indicated that patients with sarcopenic obesity and sarcopenia who received immune checkpoint inhibitors (ICIs) exhibited poor overall survival in lung cancer. In clinical practice, for patients undergoing immunotherapy, it is imperative to assess muscle mass/function and identify sarcopenic obesity/sarcopenia at the earliest opportunity. For such patients, more attention in advanced care planning and supportive care is essential to prevent a worsened prognosis. Additional research is warranted to investigate and validate effective intervention strategies aimed at improving muscle mass loss and enhancing clinical outcomes in patients with sarcopenic obesity and sarcopenia who receive ICIs.

## Introduction

Lung cancer is the frequently diagnosed cancers worldwide with an estimated 2.2 million new cases.^1,2^ Moreover, many patients are diagnosed at advanced stages with limited treatment options.^3^ The 5-year survival rate for lung cancer is significantly lower than that for other tumors. For non-small cell lung cancer (NSCLC), the 5-year survival rate is only 25%.^4^ In recent years, immune checkpoint inhibitors (ICIs) targeting the programmed death-1 (PD-1) axis and programmed death ligand-1 (PD-L1) have been clinically beneficial in patients with lung cancer.^5^ In the CheckMate 057 trial, nivolumab was associated with a higher overall survival (OS) and progression-free survival (PFS) than those associated with chemotherapy.^6^ The KEYNOTE-042 study showed that first-line pembrolizumab treatment significantly prolonged OS in patients with locally advanced or metastatic NSCLC, with better safety than that of standard chemotherapy.^7^ Although a growing number of studies have shown that patients with lung cancer can benefit from immunotherapy, the evidence is limited to a subset of patients. In patients with lung cancer, the efficiency of immunotherapy is very different, and most patients experience disease progression while undergoing treatment or after treatment discontinuation.^8^ Some established biomarkers, such as PD-L1 expression and tumor mutation burden (TMB), have proven to be powerful predictors of the effect of PD-1/PD-L1 antibodies.^9^ However, in clinical practice, many patients undergo immunotherapy without undergoing genetic testing. Assessment of TMB is difficult because of the analytical techniques involved.^10^ Therefore, a good and easily obtained model to estimate therapeutic efficacy is required.

Body composition refers to the proportion and distribution of muscle and fat tissue in the body, which plays a vital role in cancer prognosis.^11^ Sarcopenia is common in patients with cancer, and loss of skeletal muscle mass is the main feature of cancer cachexia. Studies have found that the prevalence of sarcopenia in patients with cancer has reached 38.6%, which is considerably greater than that in the general population.^12^ Moreover, muscle loss before treatment is directly associated with prognosis.^13^ In gastric cancer, lymphoma, and other tumors, the survival of patients with low muscle mass before treatment is significantly short.^14-16^ In addition, muscle loss during treatment affects patient prognosis and significantly increases the rate of treatment-related side effects significantly.^17^ Adipose tissue loss has also been shown to be associated with the clinical outcomes of cancer.^18,19^ Sarcopenic obesity (SO) is defined as low muscle mass/function and excess adiposity in the body^20^ and has been shown to be closely related to survival prognosis.^21^ Therefore, we hypothesized that body composition, sarcopenia and SO may also affect the efficacy of immunotherapy in lung cancer.

Several studies have explored the effects of muscle loss on the efficacy of immunotherapy in lung cancer. Cong et al. showed that sarcopenia is a predictor of poor survival in patients with advanced lung cancer treated with ICIs.^22^ A meta-analysis of 13 studies indicated an association between low skeletal muscle index (SMI) and reduced OS.^23^ However, some studies have investigated the effect of body fat mass on the efficacy of lung cancer immunotherapy with inconsistent conclusions.^24,25^ Additionally, only one study has investigated the association between SO, evaluated using bioelectrical impedance analysis (BIA), and outcomes in patients with lung cancer receiving ICIs. Therefore, we conducted this retrospective study to explore the potential effect of body composition on the efficacy of ICIs. In this study, the body composition was precisely defined using computed tomography (CT) at the L3 level. Additionally, we developed a body composition-based prediction model to predict the survival time of patients with lung cancer receiving immunotherapy.

## Methods and Materials

### Patients and study design

This was a single-center, retrospective study. The inclusion criteria were patients who (1) had pathologically confirmed lung cancer; (2) were 18–80-year-old; (3) were receiving immunotherapy with PD-1 or PD-L1 inhibitors; (4) had undergone an abdominal CT scan within 2 weeks before treatment; and (5) had complete data. Patients who (1) had completed prior immunotherapy, (2) had incomplete patient data, and (3) had multiple primary carcinomas were excluded.

This study was approved by the Research Ethics Committee of the Henan Provincial People’s Hospital (2022-220) and was conducted in accordance with the Declaration of Helsinki. As this was a retrospective study, patient consent was waived by the Research Ethics Committee of Henan Provincial People’s Hospital.

### Data Collection and Follow-Up

A total of 260 patients with lung cancer who received immunotherapy between May 2018 and October 2020 were identified using the hospital’s electronic medical records system. After meeting the inclusion and exclusion criteria, 119 patients were enrolled in this retrospective study. We retrospectively collected the basic clinical information of the patients before treatment, including sex, age, smoking status, height, weight, TNM stage, pathological type, and Eastern Cooperative Oncology Group Performance Status Scale (ECOG-PS). The specific drugs and treatment regimens of patients receiving PD-1 treatment, CT results, and last follow-up time were also collected. The final follow-up period was in September 2023. Overall survival (OS) was defined as the time between ICI therapy and death. Patients who still alive at the final follow-up time were censored.

### Body Composition Analysis

All patients underwent abdominal CT scans within 2 weeks prior to immunotherapy using GE machines. Cross-sectional CT at the level of lumbar vertebra L3 was used to determine the subcutaneous fat area (SFA), visceral fat area (VFA), and SMI using a software developed by Wenzhou Medical University (https://s.wzhealth.com/AIModelAnalysis/#/home?redirect=%2Findex). The skeletal muscle is distinguished from other tissues by threshold ranging from − 29 to 150 Hounsfield units and -140 to -50 Hounsfield units as fat area.^26,27^ Convolutional neural networks, representing deep learning algorithms, are mainly used for calculations.^28^ The SMI was calculated by squaring the height of the muscle area in the CT L3 cross-section. Currently, CT-L3-SMI is considered as an indicator of the overall muscle content of the body. According to previous research, the diagnostic criteria for sarcopenia are SMI of less than 43 cm^2^/m^2^ (BMI < 25) and < 53 cm^2^/m^2^ (BMI > 25) for men, and 41 cm^2^/m^2^ for women.^22^ Patients were diagnosed as with Sarcopenic obesity if they had a BMI > 23.9 and were diagnosed with sarcopenia.

### Statistical Analysis

Continuous variables are represented as mean and standard deviation, and categorical variables are represented as median and interquartile range (IQR). Receiver operating characteristic (ROC) curves were used to determine the optimal cutoff values for SFA and VFA. A 1:1 propensity score matching (PSM) was performed independently for age and sex using the nearest method with a caliper setting of 0.1. The Kaplan–Meier method was used to analyze survival time, and the log-rank test was used to detect statistical significance. Univariate Cox proportional hazards analyses were performed to determine significant factors in lung cancer. Factors with *P* < 0.2 in univariate analysis were included in multivariate Cox proportional hazards regression analyses. All factors in the multivariate analyses were incorporated into the nomogram to estimate the OS. According to the nomogram, patients were divided into high- and low-score groups based on the cutoff value of the nomogram scores. The performance of the nomogram was examined using the Kaplan-Meier method and log-rank test. All the significant factors *(P* < 0.2) in the multivariate Cox analyses were also used to create a nomogram. All analyses were performed using R software, version 4.3.3, and P values < 0.05 were considered to be statistically significant.

## Results

### Patient characteristics

**Figure 1:**
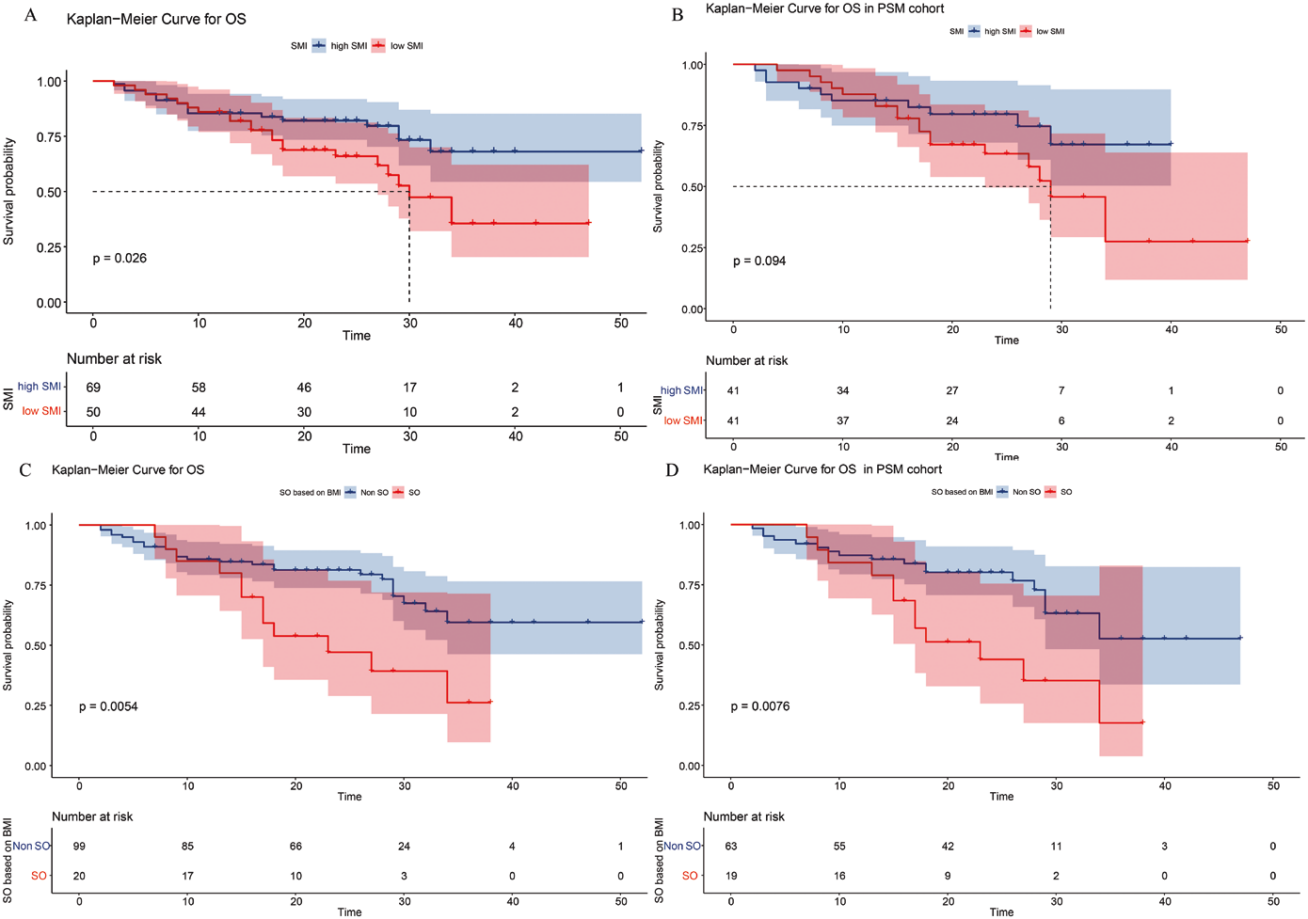
Kaplan–Meier survival curves for overall survival stratified by (A) skeletal muscle index (SMI) in the total cohort, (B) SMI in the propensity score matching (PSM) cohort, (C) sarcopenic obesity in the total cohort, (D) sarcopenic obesity in the PSM cohort.

A total of 119 patients met the inclusion criteria and were enrolled in this study. Their mean age was 60 years, mean BMI was 23.4 kg/m^2^, and 50 (42.0%) patients (23 men and 27 women) were diagnosed with sarcopenia according to our sarcopenia diagnostic criteria.^22^ Twenty (16.9%) patients were diagnosed with SO in the total cohort. Figure 1 presents the basic information of the included population, with older age in the sarcopenia population (*P* = 0.031). However, there were no significant differences in BMI, type of pathology, TNM stage, ECOG-PS, or ALB levels. In the PSM cohort, 82 patients were included with no significant differences in age and sex. The optimal SFA and VFA cutoffs were 33.11 cm^2^ and 35.82 cm^2^ with area under the curve (AUC) 54.5% and 51.6% (Table 1, Figure S1), respectively.

**Table 1:** Baseline characteristics of the study population.

		Total cohort			PSM cohort		
	level	High SMI	Low SMI	*P-value*	High SMI	Low SMI	*P-value*
N		69	50		41	41	
Sex (%)	Female	17 (24.64)	23 (46.00)	**0.025**	14 (34.15)	16 (39.02)	0.819
	Male	52 (75.36)	27 (54.00)		27 (65.85)	25 (60.98)	
Age (median [IQR])		61.00 [53.00, 69.00]	66.50 [57.00, 71.75]	**0.031**	65.00 [53.00, 70.00]	65.00 [54.00, 71.00]	0.606
(%)	> 60	35 (50.72)	33 (66.00)	0.140	24 (58.54)	26 (63.41)	0.821
	≤ 60	34 (49.28)	17 (34.00)		17 (41.46)	15 (36.59)	
BMI (mean (SD))		23.782 (2.998)	22.840 (3.328)	0.110	23.24 (3.11)	23.40 (3.16)	0.818
(%)	≥ 18.5	65 (94.20)	45 (90.00)	0.614	38 (92.68)	39 (95.12)	0.999
	< 18.5	4 (5.80)	5 (10.00)		3 (7.32)	2 (4.88)	
Smoking (%)	No	30 (44.78)	27 (55.10)	0.362	19 (46.34)	19 (47.50)	0.999
	Yes	37 (55.22)	22 (44.90)		22 (53.66)	21 (52.50)	
Types of Pathology (%)	NSCLC	56 (81.16)	43 (86.00)	0.654	34 (82.93)	35 (85.37)	0.999
	SCLC	13 (18.84)	7 (14.00)		7 (17.07)	6 (14.63)	
TNM stage (%)	Ⅱ~Ⅲ	16 (23.19)	12 (24.00)	0.999	10 (24.39)	10 (24.39)	0.999
	Ⅳ	53 (76.81)	38 (76.00)		31 (75.61)	31 (75.61)	
T (%)	1	9 (13.04)	5 (10.00)	0.051	4 (9.76)	5 (12.20)	0.161
	2	18 (26.09)	6 (12.00)		11 (26.83)	5 (12.20)	
	3	13 (18.84)	13 (26.00)		7 (17.07)	10 (24.39)	
	4	24 (34.78)	26 (52.00)		16 (39.02)	21 (51.22)	
	X	5 (7.25)	0 (0.00)		3 (7.32)	0 (0.00)	
N (%)	0	1 (1.45)	0 (0.00)	0.336	0 (0.00)	0 (0.00)	0.335
	1	8 (11.59)	4 (8.00)		4 (9.76)	4 (9.76)	
	2	33 (47.83)	31 (62.00)		18 (43.90)	24 (58.54)	
	3	24 (34.78)	15 (30.00)		17 (41.46)	13 (31.71)	
	X	3 (4.35)	0 (0.00)		2 (4.88)	0 (0.00)	
M (%)	0	16 (23.19)	14 (28.00)	0.702	10 (24.39)	12 (29.27)	0.803
	1	53 (76.81)	36 (72.00)		31 (75.61)	29 (70.73)	
ECOG-PS (mean (SD))		2.145 (0.522)	2.260 (0.527)	0.240	2.17 (0.54)	2.20 (0.51)	0.835
(%)	≥ 2	65 (94.20)	48 (96.00)	0.986	38 (92.68)	39 (95.12)	0.999
	< 2	4 (5.80)	2 (4.00)		3 (7.32)	2 (4.88)	
Surgery (%)	No	60 (86.96)	46 (92.00)	0.567	35 (85.37)	37 (90.24)	0.736
	Yes	9 (13.04)	4 (8.00)		6 (14.63)	4 (9.76)	
Type of ICIs (%)	PD-1	67 (97.10)	46 (92.00)	0.406	40 (97.56)	40 (97.56)	0.999
	PD-L1	2 (2.90)	4 (8.00)		1 (2.44)	1 (2.44)	
ALB (median [IQR])		40.80 [38.20, 43.20]	38.95 [35.30, 42.50]	0.068	40.30 [35.00, 43.20]	39.20 [35.80, 42.60]	0.572
(%)	≥ 35 g/L	57 (82.61)	41 (82.00)	0.999	31 (75.61)	35 (85.37)	0.403
	< 35 g/L	12 (17.39)	9 (18.00)		10 (24.39)	6 (14.63)	
SMI (mean (SD))		51.29 (6.40)	39.97 (5.39)	<0.001	49.82 (6.28)	41.02 (5.03)	<0.0001
SFA (median [IQR])		112.43 [77.17, 143.84]	122.63 [94.91, 148.22]	0.377	99.51 [66.38, 143.33]	122.660 [97.85, 145.59]	0.144
(%)	> 33.11	66 (95.65)	43 (86.00)	0.124	38 (92.68)	37 (90.24)	0.999
	≤ 33.11	3 (4.35)	7 (14.00)		3 (7.32)	4 (9.76)	
VFA (median [IQR])		116.38 [72.85, 193.31]	95.47 [53.52, 146.16]	0.081	103.36 [57.39, 178.11]	102.11 [62.12, 169.53]	0.937
(%)	> 35.82	64 (92.75)	41 (82.00)	0.131	36 (87.80)	35 (85.37)	0.999
	≤ 35.82	5 (7.25)	9 (18.00)		5 (12.20)	6 (14.63)	

PSM, propensity score matching; IQR, interquartile range; BMI, body mass index; SMI, skeletal muscle index; SFA, subcutaneous fat area; VFA, visceral fat area; ALB, albumin, NSCLC, Non-Small Cell Lung Cancer; SCLC, Small Cell Lung Cancer; ICIs, immune checkpoint inhibitors; PD-1, programmed cell death protein 1; PD-L1, programmed cell death ligand 1; ECOG PS, Eastern Cooperative Oncology Group Performance Status Scale.

### Influence of body composition on overall survival time

**Figure 2:**
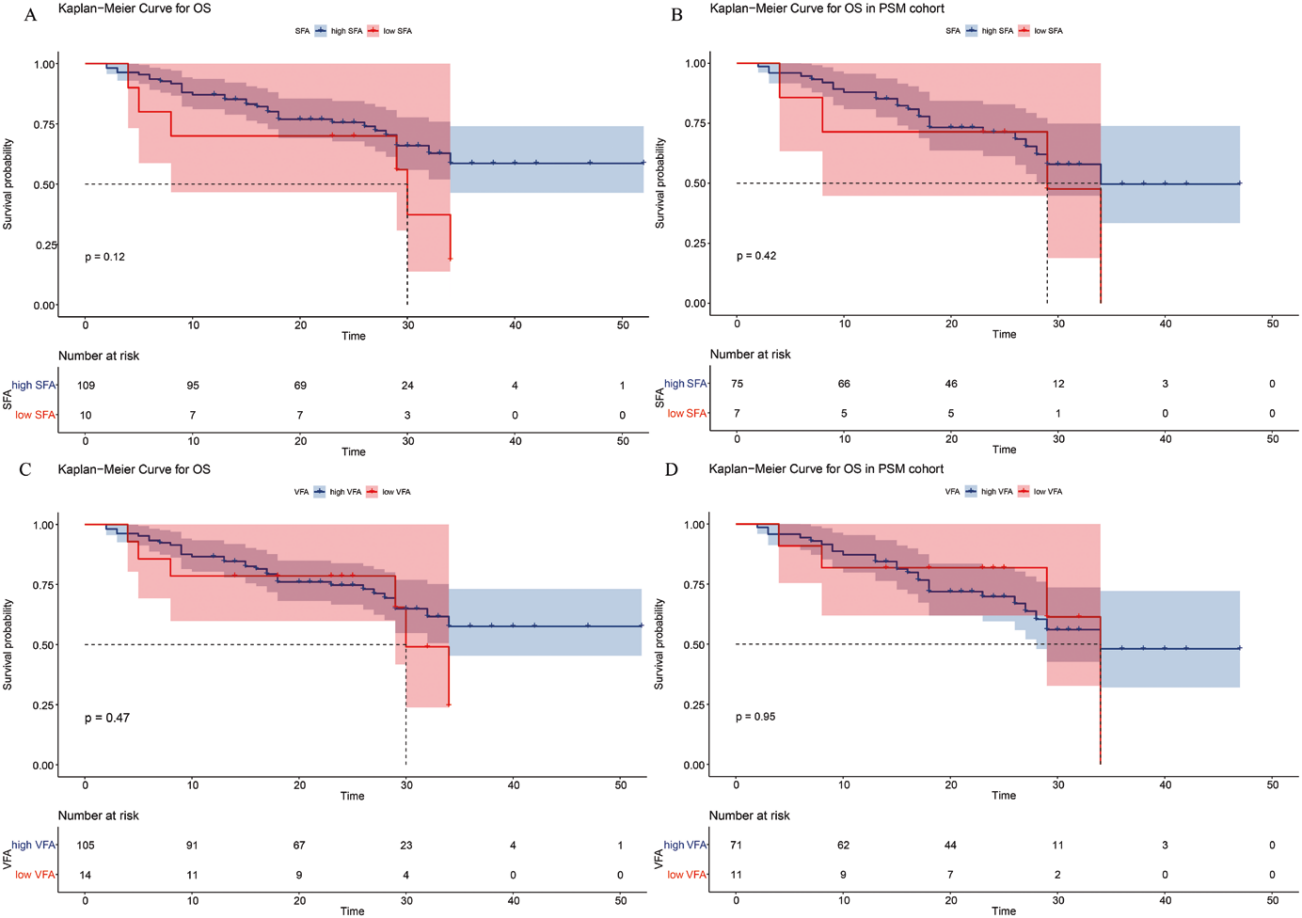
Kaplan–Meier survival curves for overall survival stratified by (A) subcutaneous fat area (SFA) in the total cohort, (B) SFA in the propensity score matching (PSM) cohort, (C) visceral fat area (VFA) in the total cohort, (D) VFA in the PSM cohort.

Kaplan-Meier analysis showed that patients with sarcopenia had a lower 3-year survival rate in the total cohort (total cohort survival rate: High SMI vs. Low SMI: 68.1% vs. 35.5%, P = 0.026). (Figure 1A) In the PSM cohort, survival analysis revealed that the low SMI group had a higher mortality rate than that of the High SMI group, but the difference was not statistically significant (High SMI vs. Low SMI: 67.2% vs. 27.5%, *P* = 0.094). (Figure 1B) In the stratification analysis by SO, SO group had higher mortality rates than those of the non-SO group. (Total cohort survival rate: SO vs. non-SO, 26.2% vs. 59.5% *P* < 0.01; PSM cohort survival rate: SO vs. non-SO, 17.6% vs. 52.6% *P* < 0.01). (Figure 1C and 1D). In addition, no significant difference in survival rate was found according to SFA (High SFA vs. Low SFA: 75.6% vs.70.0% *P* = 0.12) or VFA (High VFA vs. Low VFA: 74.8% vs. 78.6% *P* = 0.47). These results are consistent with those of the PSM cohort. (Figure 2)

Univariate Cox analysis showed that sarcopenia (*P* = 0.029), ALB ≥ 35 g/L (*P* < 0.001) and SO (*P* = 0.007) were associated with OS. SFA (HR: 1.98, 95CI%: 0.83-4.75, *P *= 0.126) and VFA (HR: 1.39, 95CI%: 0.58-3.32, *P *= 0.464) were not associated with OS. Multivariate Cox proportional hazards regression analyses revealed that sarcopenia (HR: 2.04, 95CI%: 1.04-4.01, *P* = 0.039) and SO (HR: 3.17, 95CI%: 1.49-6.75, *P* = 0.003) were also significant independent factors for OS. In the PSM cohort, sarcopenia (HR: 2.31, 95CI%: 1.01-5.27, *P* = 0.047) and SO (HR: 2.59, 95CI%: 1.18-5.66, *P* = 0.018) were still found to be significant prognostic factors for OS. (Table 2)

**Table 2:** Univariate and Multivariate analyses of overall survival in total cohort and PSM cohort

Characteristics	Total cohort				PSM cohort			
	Univariate analysis		Multivariate Cox regression analysis		Univariate analysis		Multivariate Cox regression analysis	
	HR (95% CI)	*P*	HR (95% CI)	*P*	HR (95% CI)	*P*	HR (95% CI)	*P*
**Age**								
≤ 60 vs. > 60	0.62 (0.32-1.22)	**0.165**	0.84 (0.41, 1.72)	0.637^*^	0.47 (0.21-1.06)	**0.069**	0.54 (0.23, 1.24)	0.147**
**Sex**								
Female vs. Male	0.92 (0.47-1.79)	0.798			0.93 (0.44-1.97)	0.841		
**BMI**								
< 18.5 vs. ≥ 18.5	1.25 (0.44-3.52)	0.679			0.95 (0.23-4.02)	0.947		
**Smoking**								
No vs. Yes	1.35 (0.7-2.6)	0.375			1.61 (0.74-3.5)	0.226		
**Types of Pathology**								
NSCLC vs. SCLC	1.01 (0.45-2.3)	0.973			1.23 (0.5-3.05)	0.650		
**Surgery**								
No vs. Yes	0.48 (0.12-2.02)	0.318			0.6 (0.14-2.53)	0.484		
**TNM stage**								
Ⅳ vs. Ⅱ~Ⅲ	2.05 (0.86-4.93)	**0.107**	2.20 (0.91, 5.30)	0.080^*^	2.27 (0.79-6.57)	**0.129**	2.71 (0.92, 8.00)	0.070**
**Alb level**								
≥ 35 vs. < 35	3.35 (1.7-6.61)	**< 0.001**	3.27 (1.61, 6.63)	**0.001** ^*^	2.32 (1.05-5.11)	**0.037**	2.98 (1.27, 6.98)	0.012**
**Type of ICIs**								
PD-1 vs. PD-L1	0.95 (0.23-3.97)	0.945			1.57 (0.21-11.6)	0.661		
**SMI**								
Low vs. High	2.05 (1.08-3.92)	**0.029**	2.04 (1.04, 4.01)	**0.039** ^*^	1.91 (0.89-4.13)	**0.097**	2.31 (1.01, 5.27)	0.047**
**SFA**								
Low vs. High	1.98 (0.83-4.75)	**0.126**	1.12 (0.44, 2.85)	0.814^*^	1.58 (0.55-4.56)	0.399		
**VFA**								
Low vs. High	1.39 (0.58-3.32)	0.464			0.98 (0.34-2.83)	0.974		
**Sarcopenic Obesity**								
Yes vs. NO	2.56 (1.29-5.10)	**0.007**	3.17 (1.49-6.75)	**0.003** ^**#**^	2.66 (1.26-5.58)	**0.010**	2.59 (1.18-5.66)	**0.018** ^**##**^

PSM, propensity score matching; CI, confidence interval; HR, hazard ratio; BMI, body mass index; NSCLC, Non-Small Cell Lung Cancer; SCLC, Small Cell Lung Cancer; PD-1, programmed cell death protein 1; PD-L1, programmed cell death ligand 1; SMI, skeletal muscle index; SFA, subcutaneous fat area; VFA, visceral fat area; ALB, albumin.

^*^: adjusted for age, TNM stage, Alb level, SMI and SFA; #: adjusted for age, TNM stage, Alb level, SFA and Sarcopenic Obesity; **: adjusted for age, TNM stage, Alb level and SMI; ##: adjusted for age, TNM stage, Alb level and Sarcopenic Obesity.

### Development of a nomogram model for OS

**Figure 3:**
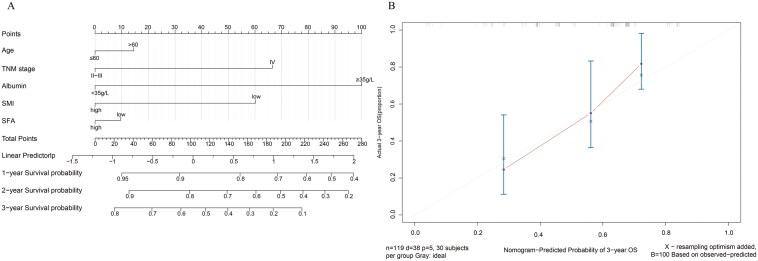
Nomogram (A) established based on body composition, age, TNM stage and albumin for predicting overall survival time in patients with lung cancer treated with immune checkpoint inhibitors. (B) The calibration curves in the total cohort.

A nomogram was established to predict OS in patients with lung cancer treated with ICIs. The nomogram incorporated all factors in the multivariate analyses, including SMI, age, TNM stage, ALB level, and SFA. A calibration curve was constructed for the nomogram to determine its predictive ability. The calibration curve closely resembled the outcomes observed for the entire cohort. The C-index of the nomogram was 0.685. These results suggest that the nomogram performed satisfactorily. (Figure 3) The nomogram based on the significant factors, including SMI, age, TNM stage and ALB level were also created and presented in the supplemental files.(Figure S2)

### Predictive of value of nomogram

**Figure 4:**
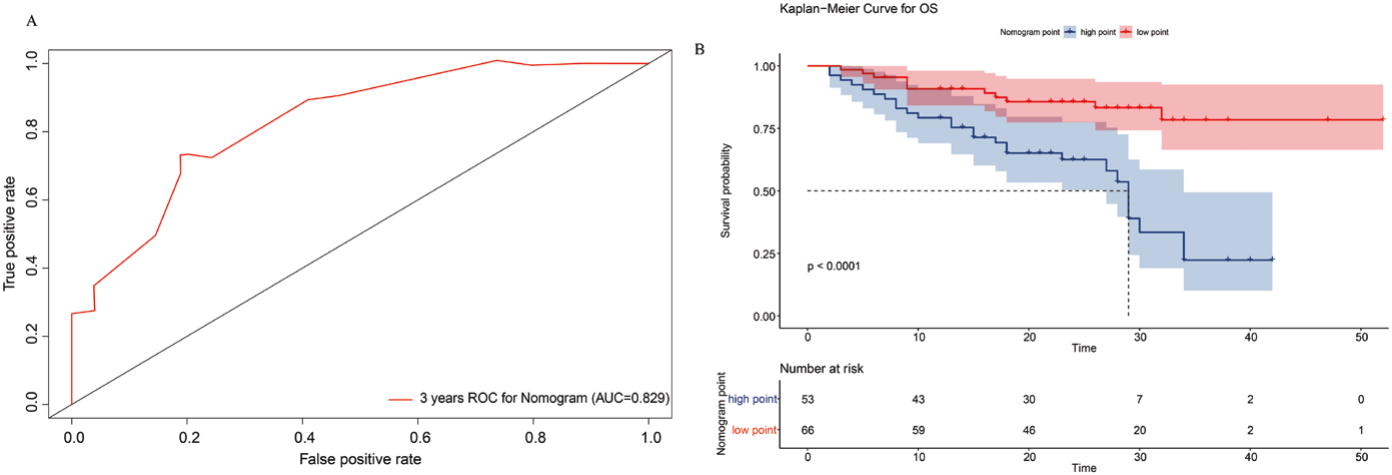
Time-dependent ROC curves(A) for survival prediction for nomogram scores. (B) Kaplan–Meier survival curves for overall survival stratified by nomogram scores.

The optimal cutoff value of the nomogram score (193.44) was determined using the ROC curve (Figure 4A). Based on the cutoff value, 53 patients were assigned to the high-score group and 66 to the low-score group. The Kaplan–Meier analysis of these two groups is presented in Figure 4B. Patients in the high-score group had shorter survival time than those in the low-score group. (*P* < 0.0001)

## Discussion

This study explored the predictive value of body composition for the survival of patients with lung cancer treated with ICIs. Patients with low skeletal muscle mass treated with immunotherapy had a significantly shorter OS than those without sarcopenia. An increased mortality rate has been observed in patients diagnosed with SO. Moreover, fat mass was not associated with mortality in patients with lung cancer who received ICIs. In addition, we created a nomogram that incorporated body composition and other factors to predict the survival of patients with lung cancer receiving immunotherapy. This model has also been validated as a simple tool for predicting individualized survival.

In the present study, we showed that sarcopenia is a potential predictor of OS in patients with lung cancer receiving ICIs. Our results are consistent with those of previous studies. In a study of 105 patients with non-small cell lung cancer, sarcopenia was a predictor poor prognosis.^29^ Another study showed that patients with NSCLC and sarcopenia have lower overall response rate.^30^ However, the results of some studies have been inconsistent. A low PMI defined by CT did not have a significant impact on OS according to a study conducted in Japan.^31^ The differences in results may be mainly due to the different methods of measuring muscle mass using CT and the different diagnostic criteria for sarcopenia. In our study, a tool to measure muscle area using convolutional neural networks was utilized to measure skeletal mass and fat mass at the L3 level, which reduced measurement errors.^28^ In obese patients, muscle loss is often overlooked.^11^ An increasing number of studies have found that SO can significantly affect the incidence of complications in patients with cancer, and further affect their prognoses.^32,33^ To date, only one study has been conducted on SO in patients with lung cancer. Hui-Ping Ding et al. reported a significant correlation between SO and survival in patients with NSCLC treated with immunotherapy. However, SMI was measured using BIA, which is less accurate.^34^ This is the first study to investigate the association between SO measured using CT and OS in patients with lung cancer treated with ICIs.

Alterations in body composition in patients with cancer are common and negatively affect clinical outcomes. Sarcopenia is one of the major manifestations of changes in body composition in patients with cancer. The prevalence of sarcopenia in patients with lung cancer ranges from 43% to 52%.^35^ Sarcopenia is also been shown to be a significant negative prognostic factor in patients with lung cancer treated with chemotherapy, radiotherapy, and surgery. A prospective cohort study of 64 patients with lung adenocarcinoma and synchronous bone metastases showed that sarcopenia was associated with poor survival (HR = 2.96).^36^ In patients with NSCLC undergoing stereotactic body radiotherapy (SBRT), low skeletal muscle mass is a significant contributor to mortality.^37^ Additionally, several studies have shown that sarcopenia is associated with poor survival outcomes in patients treated with ICIs.^38,39^ The mechanisms underlying the relationship between sarcopenia and prognosis after immunotherapy remain unclear. Many studies have found that chronic inflammation plays a potential role in influencing the efficacy of immunotherapy. Muscle loss can lead to increased systemic inflammation, such as an elevated neutrophil-to-lymphocyte ratio (NLR) and CRP level, subsequently reducing the effectiveness of immunotherapy.^40,41^ Additionally, skeletal muscle cells affect the efficacy of immune treatments by secreting IL-15 and regulating circulating natural killer cells and CD8 + T cells.^42,43^ Muscle loss can result in immune escape and impaired immune function, thereby affecting the effectiveness of ICI drugs.^44^ Finally, sarcopenia is also a manifestation of malnutrition and cancer cachexia, which can also seriously affect the efficacy of immunotherapy and increase the incidence of adverse reactions.^45,46^

Importantly, our results revealed that obese or overweight patients with sarcopenia have the worst prognosis. However, the mechanism by which SO affects tumor prognosis remains unclear. On the one hand, patients with SO have poor tolerance to treatment. On the other hand, higher body fat content in patients with SO leads to increased exposure to some therapeutic drugs, which in turn increases the toxic effects of treatment.^11^ Published studies have shown that SO to be independently related to grade 2-4 neurotoxicity and dose-limiting toxicity during cycle one of chemotherapy for esophageal cancer.^47,48^ Considering that SO is difficult to diagnose, more studies are required to explore the diagnostic methods for SO and the mechanism underlying its impact on the efficiency of ICIs.

Notably, no significant difference was observed between low VFA/SFA and prognosis in patients with lung cancer treated with ICIs. In the previous study, high SFA have been shown to be associated with favorable OS in patients with metastatic renal cancer treated with anti-PD1.^49^ One study found that patients with lung cancer with high subcutaneous fat mass who received immunotherapy had a longer OS.^24^ However, the results of our study and those of others are contrasting with these results,^25^ suggesting that more trials are required to further verify the prognostic role of fat mass in ICI efficiency.

Our study had some limitations. First, this was a single-center, retrospective study. Although PSM analysis was used, bias in our results cannot be ignored. Second, the use of different treatment regimens in the study population may have contributed to the heterogeneity of the results. Third, some prognostic biomarkers such as TMB and PD-L1 expression were not clinically tested and were not included in the analysis. Fourth, muscle strength, another dimension of sarcopenia, was not evaluated. Fifth, as this was a retrospective study, it was impossible to explore the potential impact of sarcopenia on PFS. Finally, the sample size was small.

## Conclusion

In conclusion, SO and sarcopenia predicted poor overall survival in patients with lung cancer who received ICIs. Before immunotherapy, more attention should be paid to the patient’s body composition to identify sarcopenia. The prognostic significance of body composition assessment can enhance advanced care planning and facilitate the communication of prognostic information to patients and their families. Further studies are required to confirm the impact of sarcopenia and SO on the efficiency of immunotherapy. Further research is needed to focus on various intervention strategies, including exercise, nutritional, and pharmacological interventions, to enhance clinical outcomes in patients with SO and sarcopenia.

## Supplementary Material

oyaf114_suppl_Supplementary_Figures_S1-S2

## Data Availability

The data that support the findings of this study are available from the corresponding author, upon reasonable request.
