# Supplementary material for: The prognostic value of sarcopenia and sarcopenic obesity in patients with lung cancer receiving immunotherapy: a propensity score matching study
Source: Oncologist. 2025 Jun 23;30(6):oyaf114. doi: 10.1093/oncolo/oyaf114 (PMC12205993; doi:10.1093/oncolo/oyaf114)
Supplement: oyaf114_suppl_Supplementary_Figures_S1-S2 [file oyaf114_suppl_supplementary_figures_s1-s2.docx]

Supplemental files


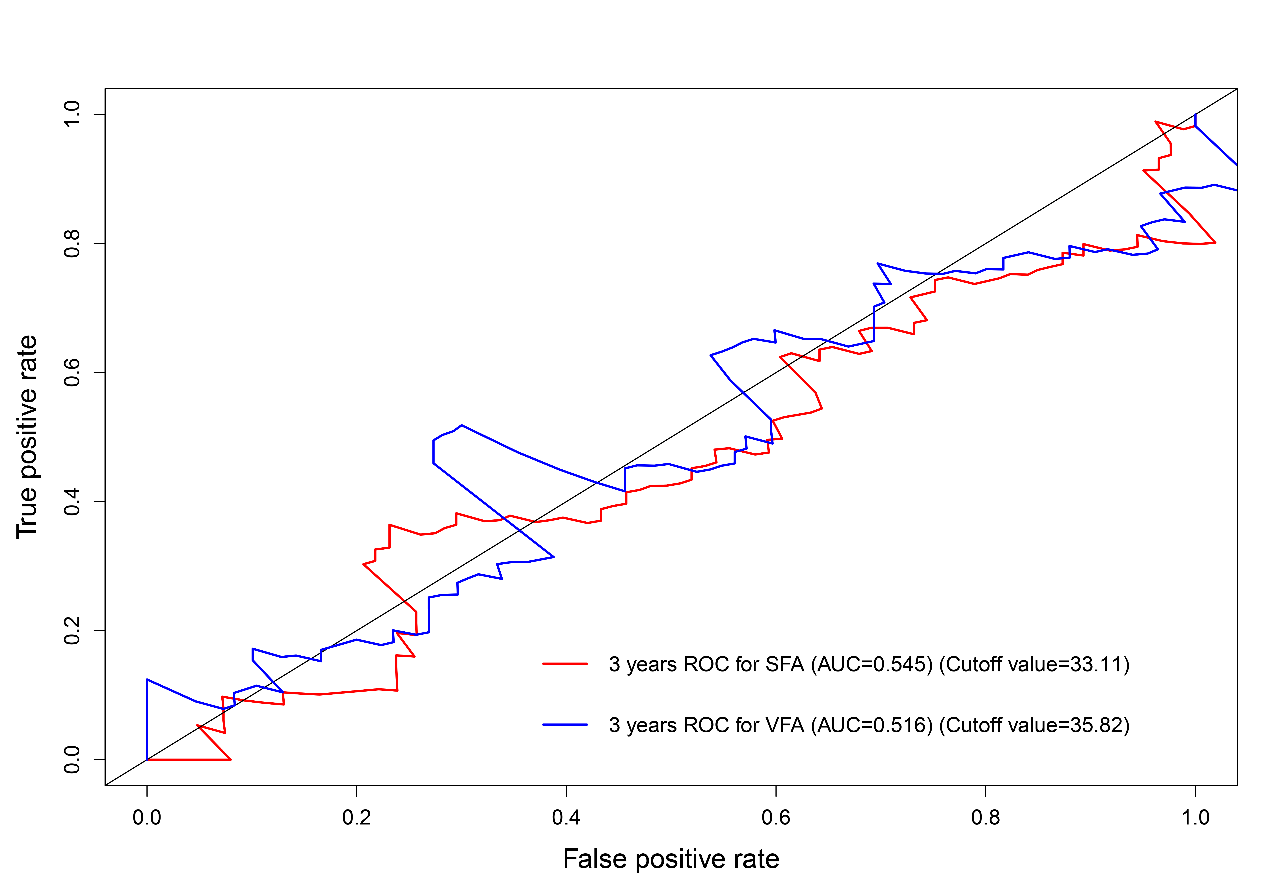


Figure S1 Time-dependent ROC curves for survival prediction for subcutaneous fat area (SFA) and visceral fat area (VFA).


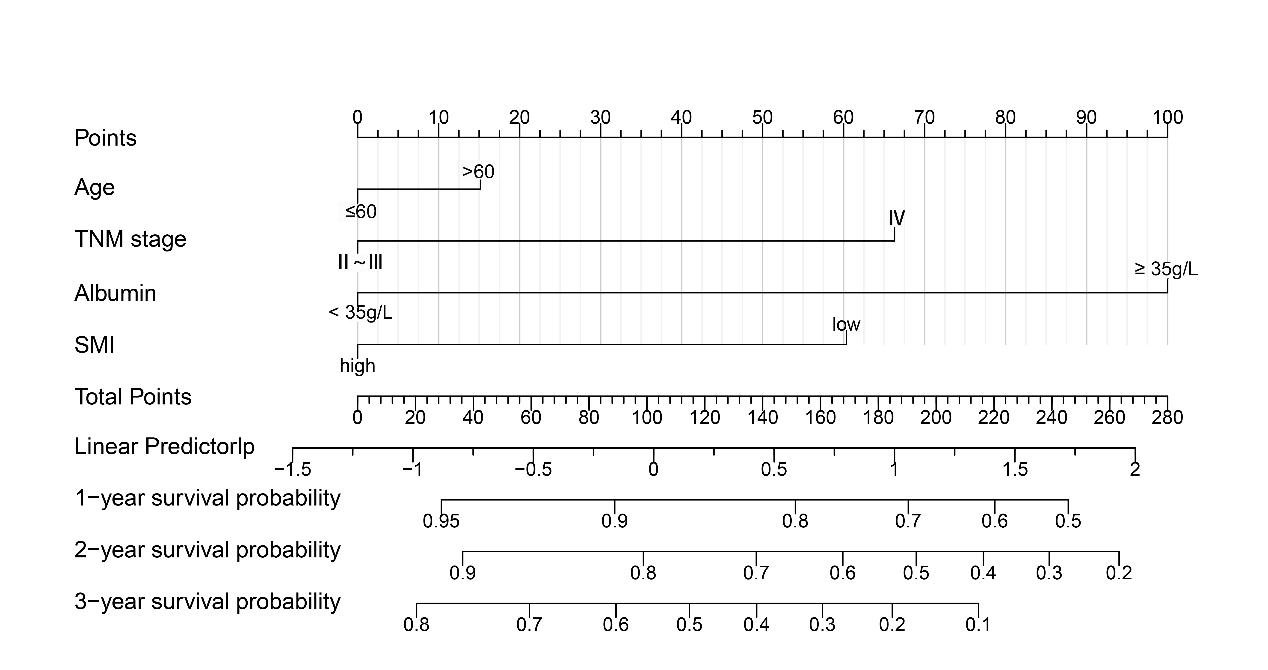


Figure S2 Nomogram established based on significant factors (p < 0.05) for predicting overall survival time in patients with lung cancer treated with immune checkpoint inhibitors.
